# Supplementary figures and images for: Respiration modulates oscillatory neural network activity at rest
Source: PLoS Biol. 2021 Nov 11;19(11):e3001457. doi: 10.1371/journal.pbio.3001457 (PMC8610250; doi:10.1371/journal.pbio.3001457)

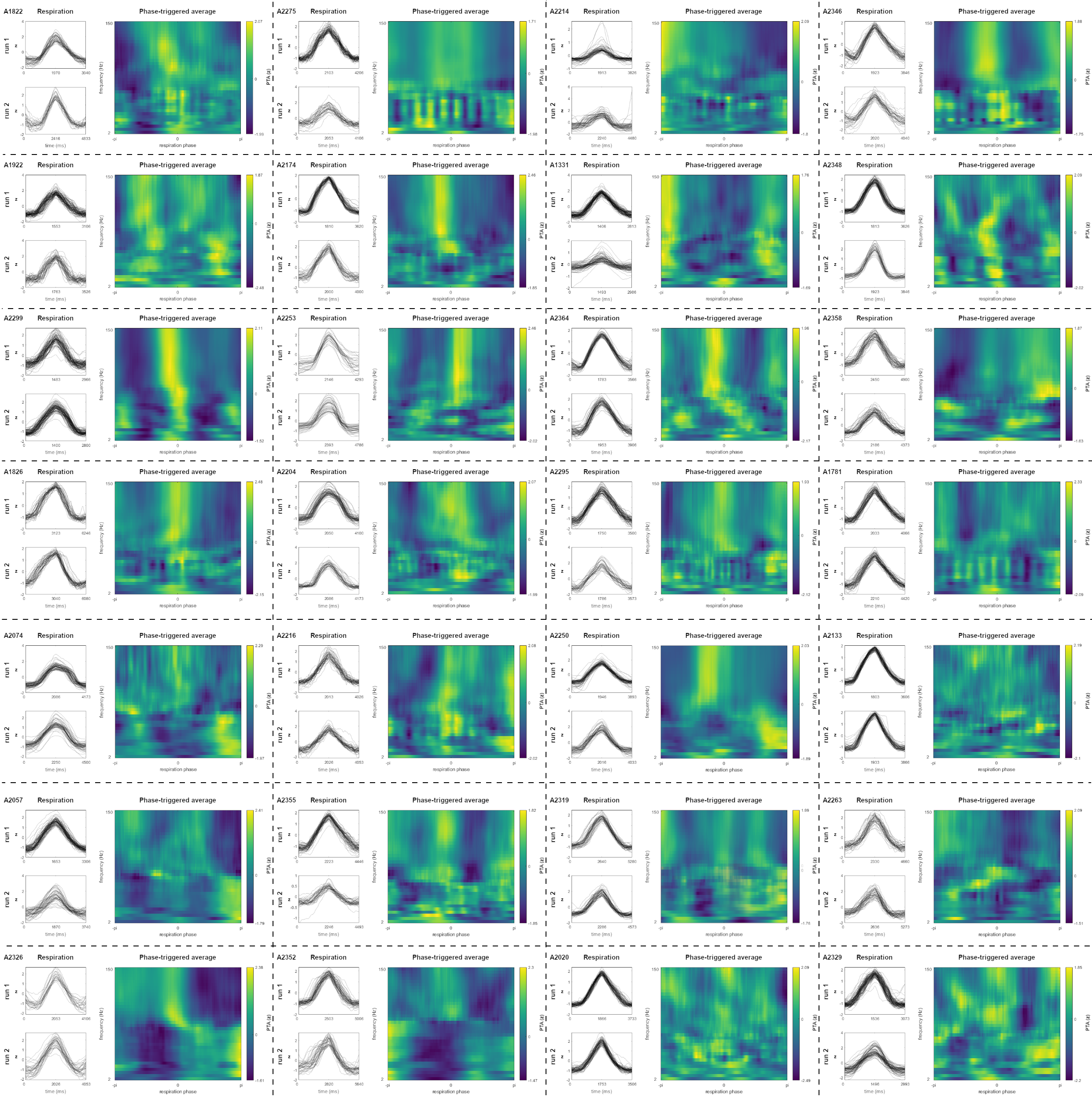

Supplement: S2 Fig — Left panels show single respiration traces centred around peak inspiration from each run. Right panel shows the individual phase–amplitude spectrogram averaged across all sensors. PTA values are shown as z-values, i.e., normalised within each frequency to reveal phase-related modulations. Underlying data are provided in the folder “Supplementary Information” on the OSF directory. PTA, phase-triggered average. (TIF) [file pbio.3001457.s003.tif]

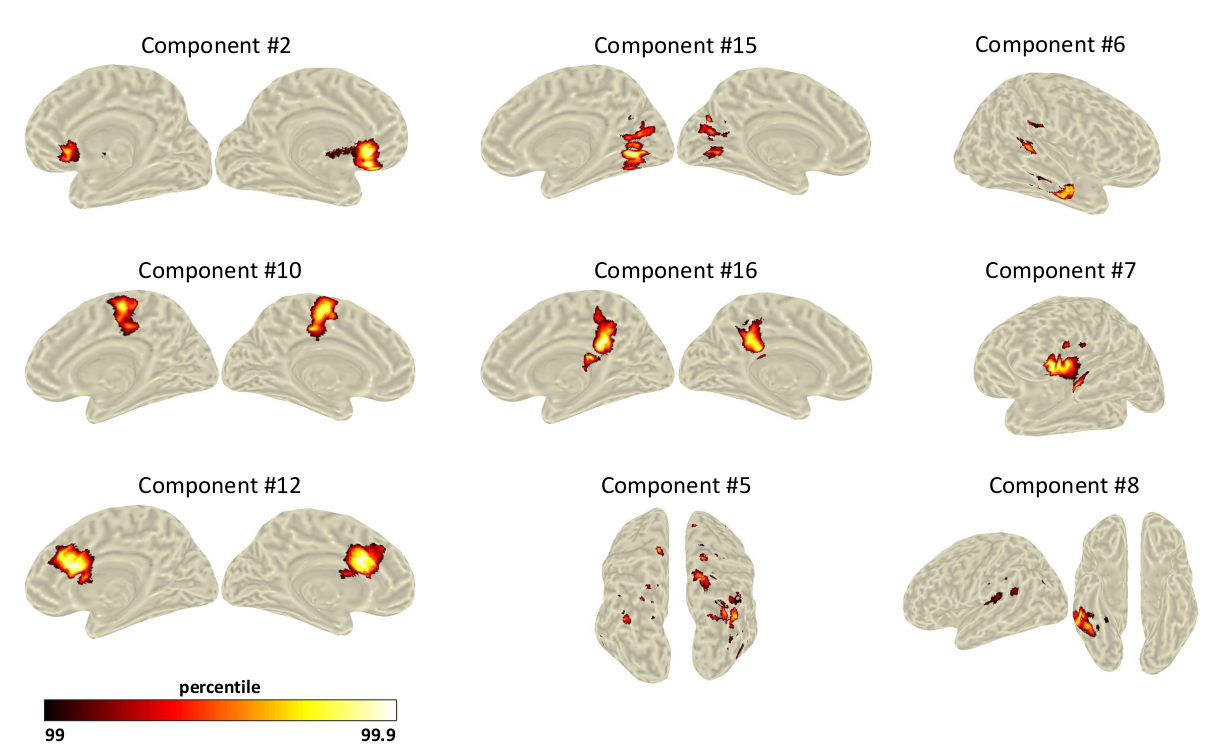

Supplement: S3 Fig — As in Fig 2, whole-brain NMF components were thresholded at the 99th percentile, resulting in anatomical locations with an extent of n = 202 voxels. Underlying data are provided in the folder “Supplementary Information” on the OSF directory. NMF, nonnegative matrix factorisation. (TIF) [file pbio.3001457.s004.tif]

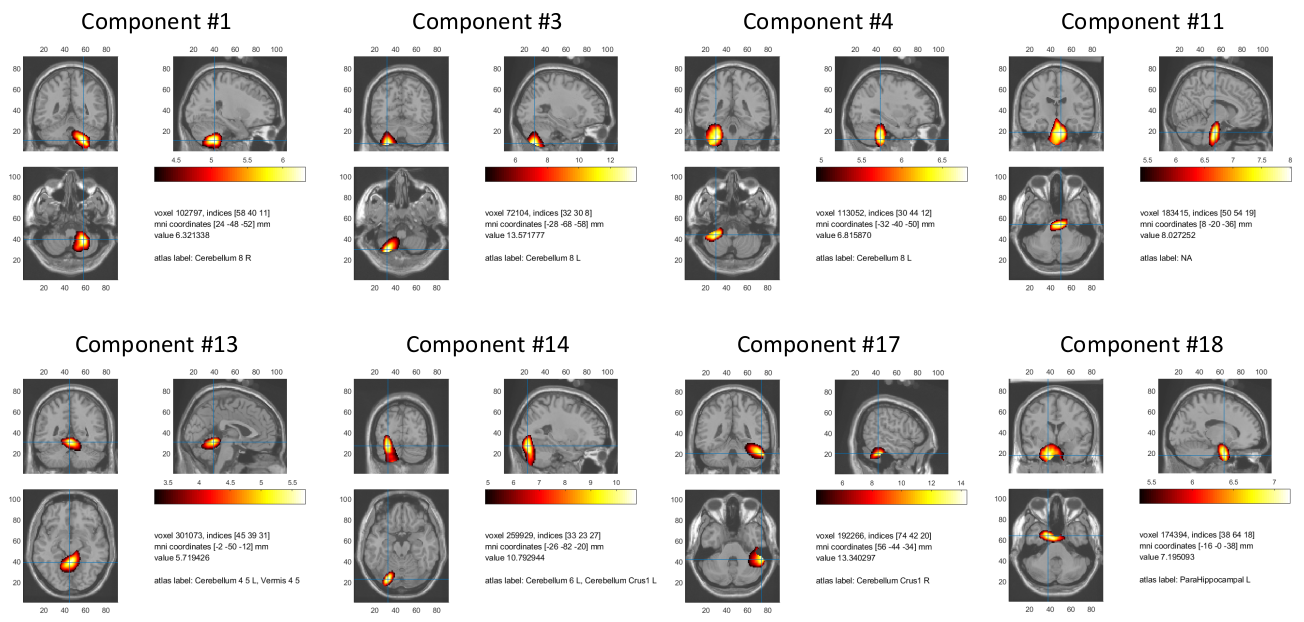

Supplement: S4 Fig — As in Fig 2, whole-brain NMF components were thresholded at the 99th percentile, resulting in anatomical locations with an extent of n = 202 voxels. Crosshairs are positioned at the peak voxel of each location, and atlas labels are provided (corresponding to the nomenclature in Fig 2B). Underlying data are provided in the folder “Supplementary Information” on the OSF directory. NMF, nonnegative matrix factorisation. (TIF) [file pbio.3001457.s005.tif]

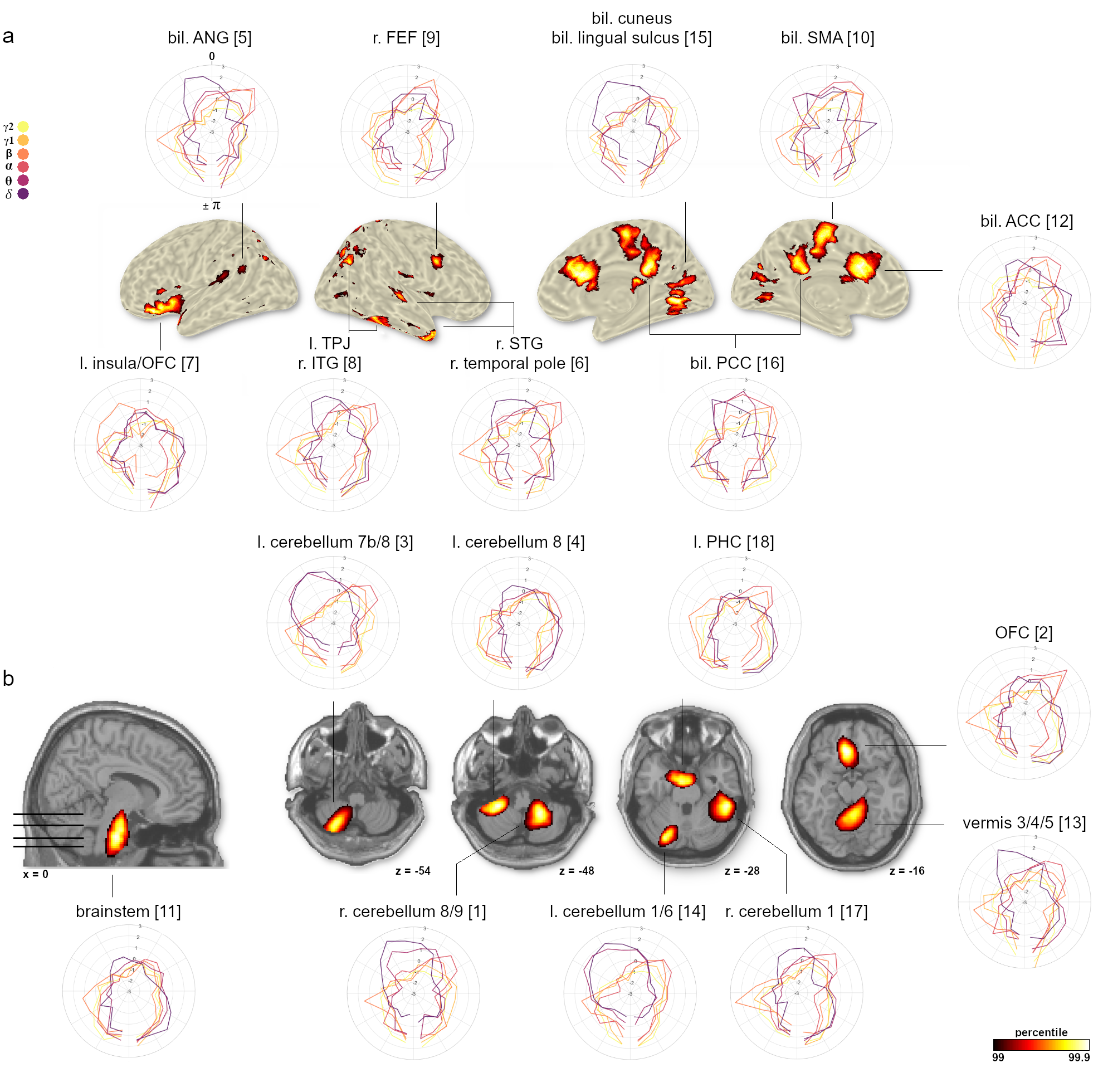

Supplement: S5 Fig — (A) Cortical components plotted on an inflated brain surface. Polar plots show group-level normalised MI time courses averaged within frequency bands (delta to high gamma) over the entire respiration cycle. (B) Subcortical components plotted on transverse and sagittal slices of the MNI brain. Same format as A. Underlying data are provided in the folder “Supplementary Information” on the OSF directory. MI, modulation index; MNI, Montreal Neurological Institute; NMF, nonnegative matrix factorisation. (TIF) [file pbio.3001457.s006.tif]

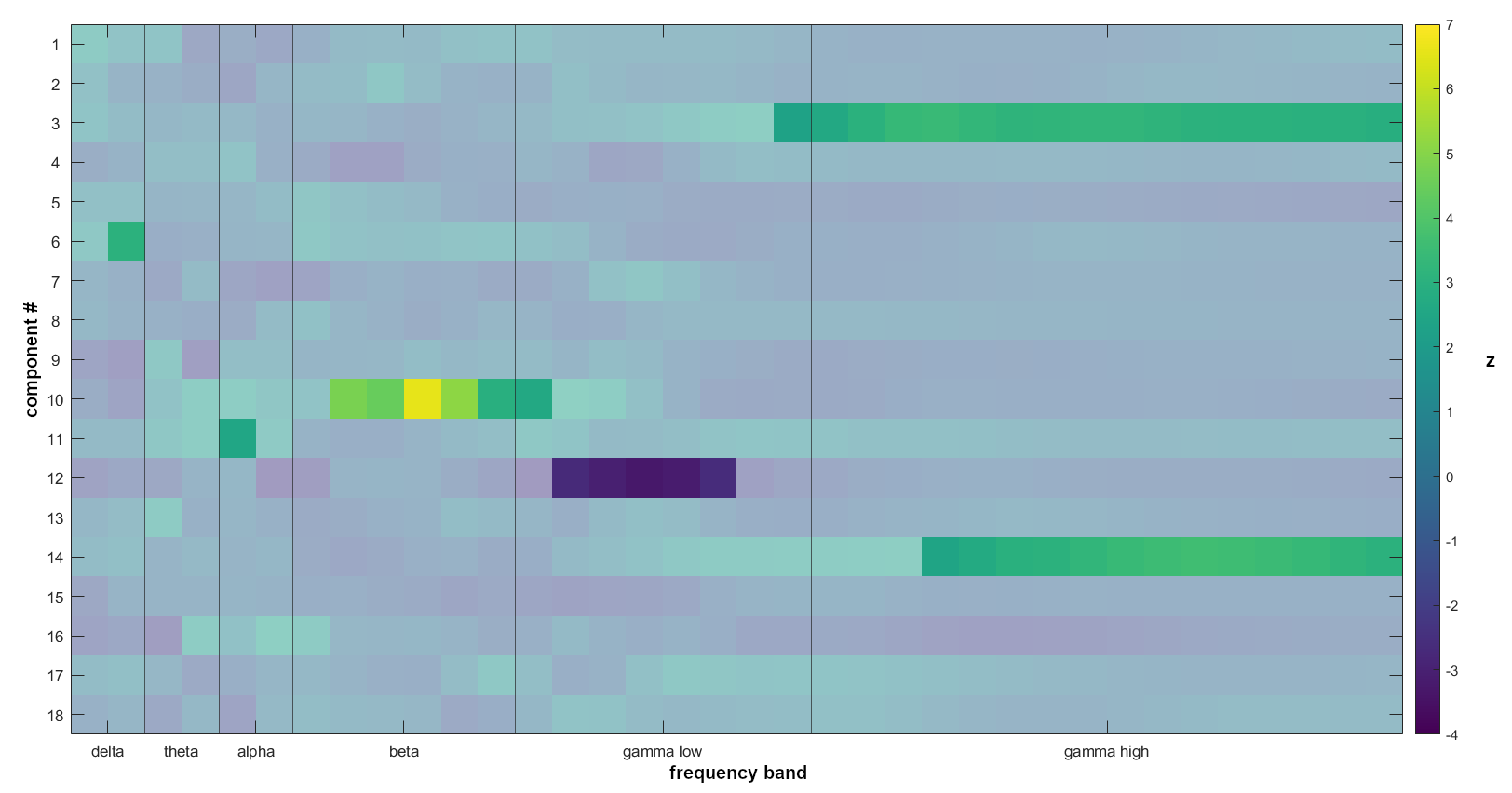

Supplement: S6 Fig — For each component and each frequency, we computed z-scores by comparing frequency-specific MI values of a particular component to the average across all other components (see main text for details). Opacity indicates significant differences (i.e., z = ± 2.33). Underlying data are provided in the folder “Supplementary Information” on the OSF directory. MI, modulation index. (TIF) [file pbio.3001457.s007.tif]

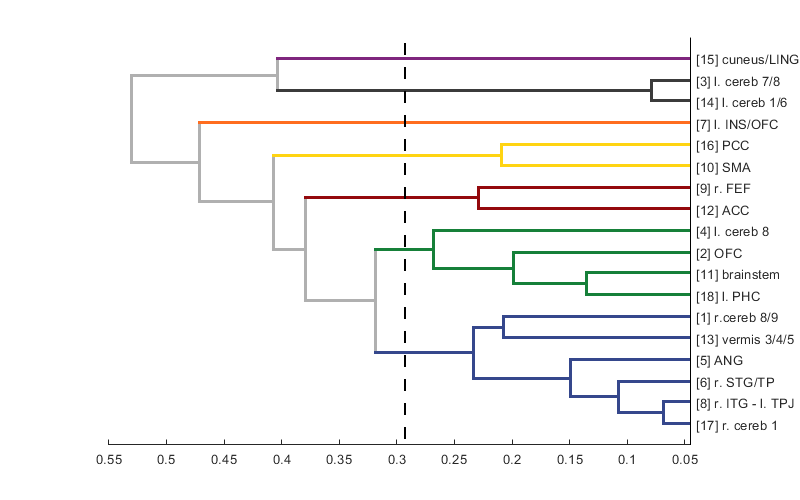

Supplement: S7 Fig — Dashed vertical line illustrates the cutoff criterion, yielding a total of 7 clusters. Cluster colouring is identical to Figs 3 and 4. Underlying data are provided in the folder “Supplementary Information” on the OSF directory. NMF, nonnegative matrix factorisation. (TIF) [file pbio.3001457.s008.tif]

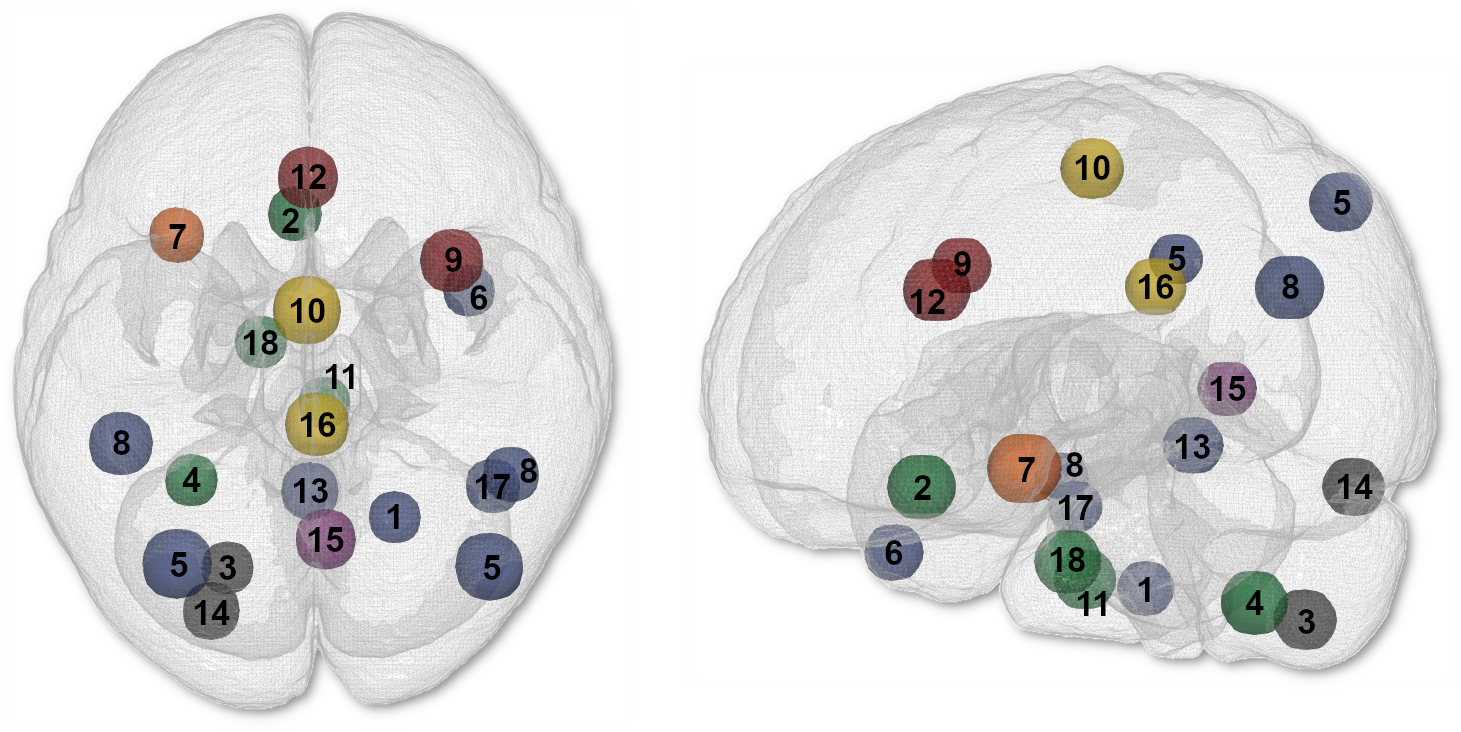

Supplement: S8 Fig — Numbering corresponds to Figs 2 and 4C as well as S3 and S4 Figs. Cluster colouring is identical to Figs 3 and 4 as well as S7 Fig. Underlying data are provided in the folder “Supplementary Information” on the OSF directory. NMF, nonnegative matrix factorisation; RMBO, respiration-modulated brain oscillation. (TIF) [file pbio.3001457.s009.tif]

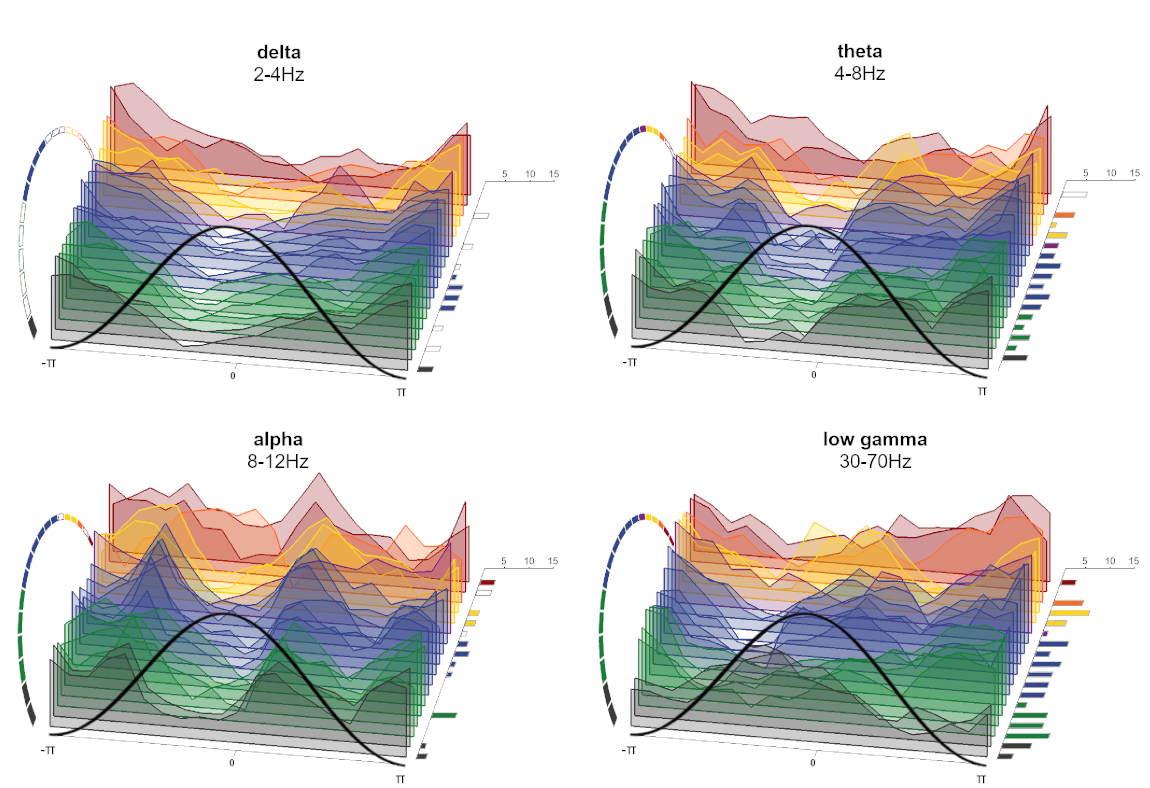

Supplement: S9 Fig — Clusters of NMF components are shown in the same order as in Fig 4C. Right panel bar graphs show the number of participants whose modulation within the respective component was strongest for the depicted frequency band (versus all other frequency bands). Coloured bars and circular segments mark NMF components for which the respective frequency band was significantly modulated by respiration phase. Underlying data are provided in the folder “Supplementary Information” on the OSF directory. NMF, nonnegative matrix factorisation. (TIF) [file pbio.3001457.s010.tif]

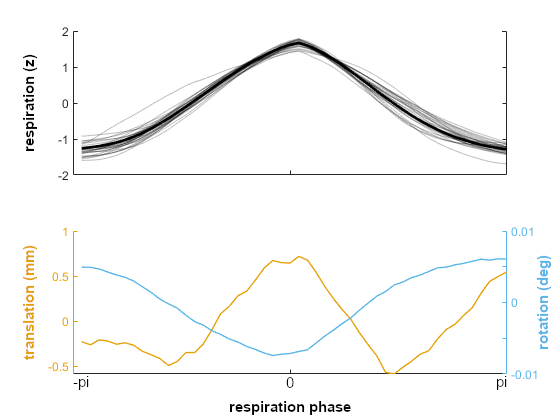

Supplement: S10 Fig — Top panel shows individual (grey lines) and group-level average time courses of the normalised respiration signal (bold). Bottom panel shows group-level average head movement signals phase locked to the respiration signal. Both translation (measured as Euclidean distance, yellow) and rotation (blue) are depicted as vector norms combining movement traces in x, y, and z directions. Underlying data are provided in the folder “Supplementary Information” on the OSF directory. (TIF) [file pbio.3001457.s011.tif]
