# Supplementary material for: Respiration modulates oscillatory neural network activity at rest
Source: PLoS Biol. 2021 Nov 11;19(11):e3001457. doi: 10.1371/journal.pbio.3001457 (PMC8610250; doi:10.1371/journal.pbio.3001457)
Supplement: S1 Text — LMEM, linear mixed effect model. (DOCX) [file pbio.3001457.s012.docx]

**S1 Text**

Extended LMEM analysis of spatial patterns across planes

In the main text, we investigated MI spectra as a function of distance to the head centre. As the distance measure was computed as the vector norm from x, y, and z coordinates (see Methods), we here extend the analysis of individual planes. To this end, we report frequency-specific LMEM analyses modelling individual contributions of the three planes, e.g.

MI_j_ = 𝛽_0_ + (𝛽_1_ + *S*_1j_) * *x* + (𝛽_2_ + *S*_2j_) * *y* + (𝛽_3_ + *S*_3j_) * *z +* *e*_j_

For participant j, the modulation index is expressed as a combination of the intercept (𝛽_0_), the fixed effects of the components’ coordinates in x, y, and z plane (𝛽_1,_ 𝛽_2,_ 𝛽_3_), and an error term (e_j_ ~ N(0,σ²)). We accounted for between-participant variation by specifying random slopes (S_1j,_ S_2j_ S_3j_). This way, we can gain further insight as to how each plane contributed to the overall head centre effects and how spectrally specific these effects are. The results are presented in S1 Table.
