# Supplementary material for: Respiration modulates oscillatory neural network activity at rest
Source: PLoS Biol. 2021 Nov 11;19(11):e3001457. doi: 10.1371/journal.pbio.3001457 (PMC8610250; doi:10.1371/journal.pbio.3001457)
Supplement: S2 Text — (DOCX) [file pbio.3001457.s013.docx]

**S2 Text**

Control analysis for high-frequency muscle artefacts

Given the reported modulatory effects in the gamma band, it is crucial to rule out that they were caused by artificial, high-frequency muscle (EMG) activity. To this end, we conducted an ICA in an attempt to isolate individual EMG components and repeat the analyses of MI and PTA on the time courses of these components. As our participants are carefully instructed to minimize muscle activity and oftentimes have participated in several MEG studies prior to our study, our present data (fortunately) contained only very little contamination from muscle activity (as validated independently by both authors). Thus, we were not able to extract at least one ICA component reliably representing high-frequency EMG activity for most participants, which is required in order to allow the estimation of group-level effects.

We therefore conducted a similar, but slightly adapted analysis to characterise the relationship between even minute muscle artefacts and respiration. Specifically, we high-pass filtered our data using a 4th-order (forward and reverse) Butterworth filter with a cut-off frequency of 60Hz to amplify muscle-related contaminations. We then subjected the filtered data to ICA analysis (extracting the first 50 components). Each extracted component was individually scanned for the occurrence of muscle activity using the automatised Fieldtrip *function ft_artifact_muscle* (see http://new.fieldtriptoolbox.org/reference/ft_artifact_muscle/ for documentation). This function applies an 8th-order Butterworth bandpass filter (110 - 140Hz) followed by computation of a smoothed amplitude envelope using the Hilbert transform. Muscle artefacts are then identified as events exceeding a default threshold of z = 4 in normalised data. Using this function, we determined the number of muscle artefact events within each ICA component. We defined the component with the highest number of events as the ‘artefact component’ (containing an average of 11.07 ± 4.22 artefact events, M ± SD) and the component with the lowest number of events as the ‘control component’ (0.07 ± 0.38 artefact events). Finally, we performed the same MI analysis as before (see Methods section for details) separately for each participant’s artefact and control components, respectively.

The reasoning behind computing MI for both components was as follows: In the absence of clearly identifiable movement-related ICA components in our data, the MI spectrum of the artefact component will almost inevitably reflect respiration-coupling of compound artificial and meaningful parts of the signal. Therefore, arguably the only way to isolate the contributions of movement artefacts is the contrast between the artefact component and a control component that does not contain such artefacts. If it was indeed the case that muscle activity confounded the MI results (see Results), the artificial increment within the artefact component (i.e. the difference between the two MI courses) would have to be systematically related to the respiration signal.

Consequently, we computed MI spectra of the artefact and control components for both runs of each participant. We then applied a pairwise t-test for each frequency to identify systematic MI differences between artefact and control components. Even before correcting for multiple comparisons across the frequency dimension, none of the MI difference values reached significance. The lack of significant coupling between respiration and even minute high-frequency muscle artefacts provides further evidence against a confound of muscle activity in the presented MI analyses. The lack of EMG artefacts in our data is not surprising, given that a) participants were explicitly instructed to sit relaxed and refrain from clenching their teeth or neck, blinking etc. and b) the task-free measurement made any and all muscle movements unnecessary. Furthermore, the manuscript cites a rather extensive body of literature demonstrating the link between respiration and gamma oscillations by means of phase-amplitude coupling (see Discussion).
