# Supplementary material for: Respiration modulates oscillatory neural network activity at rest
Source: PLoS Biol. 2021 Nov 11;19(11):e3001457. doi: 10.1371/journal.pbio.3001457 (PMC8610250; doi:10.1371/journal.pbio.3001457)
Supplement: S3 Text — (DOCX) [file pbio.3001457.s014.docx]

**S3 Text**

Control analysis for spectrally unspecific modulations

In order to validate our interpretation of complex, frequency-specific modulations, we conducted a control analysis to investigate whether a more general, spectrally unspecific mechanism could equally explain our effects. Conceivably, such a mechanism could depend on CO_2_-levels and would merely relate respiration to ongoing oscillatory activity within brain regions. Any temporo-spectral differences across brain regions would then simply be due to differences in activation profiles of those regions, entirely independent of the differential respiratory modulation we propose.
To exclude such an alternative explanation, we tested to what extent the modulation spectra we observed were related to ongoing power spectra (independent of respiratory modulation). To this end, we used the output of the wavelet transform to compute voxelwise power spectra for each frequency used in our main analyses (i.e., 20,173 voxels x 36 frequencies). In keeping with our general analysis pipeline, we then restricted these whole-brain power spectra to component-specific maps, i.e. to those 202 suprathreshold voxels making up a given NMF component. For each of the 18 NMF components, power spectra were averaged across voxels, yielding one mean power spectrum per component per participant. Finally, for each component, we computed the Spearman correlation between each participant’s average power spectrum and their average MI spectrum, yielding 28 correlations x 18 components. To assess consistent positive relations between power and MI spectra, each component’s correlation coefficients were Fisher- z-transformed and subsequently tested against zero with a one-sided t-test. None of the components showed a significant group-level correlation between the two spectra (all p > .077 before multiple comparison correction). In our view, these results provide further evidence against a perfectly reasonable ‘simple’ mechanism (such as mere variation in CO_2_ levels) and in contrast argue for spectrally specific modulatory effects.

Control analysis for movement-related changes in oscillatory power

In order to control for potential power changes related to breathing-induced head motion, we subjected a total of 36 GLM regression weights for each participant (2 movements [translation, rotation] x 3 directions [x, y, z] x 3 orders [first, second, third] x 2 levels [raw, derivatives]) to a group-level analysis: Individual regression weights were averaged across both runs, yielding a matrix of 36 regression weights x 20,173 voxels per participant. Applying the same cluster permutation approach we followed throughout our manuscript, we can confirm that there were indeed no clusters of voxels whose head movement regressors were significantly different from zero across participants.

There are several explanations why respiration-related head movements do not lead to systematic power changes at the group level. First, head movements are individually different, both those that are related to respiration and those that are unrelated to respiration.

Second, not all head movements are expected to lead to power changes in the signal. Our voxel-level analysis utilises adaptive beamformers that are based on a linear combination of all sensor signals. Several movements (such as rotations, lateral movements, or movements along the posterior-anterior axis) will lead to simultaneous power increase in some sensors, but a decrease in others with small (or none) net power change.

Third, S10 Fig illustrates that respiration-related movements are very small (especially in comparison to other head movements such as the typical ‘sinking’ of participants across the duration of the measurement). This also explains why movement effects on power are not consistent across participants.
