# Supplementary material for: Respiration modulates oscillatory neural network activity at rest
Source: PLoS Biol. 2021 Nov 11;19(11):e3001457. doi: 10.1371/journal.pbio.3001457 (PMC8610250; doi:10.1371/journal.pbio.3001457)
Supplement: Attachment — Submitted filename: REST_PLOS_R1_211005_Resp2Refs.docx [file pbio.3001457.s015.docx]

**Reviewer #1:** The manuscript by Kluger and Gross analyzes the effect of respiration on oscillatory brain activity in humans at rest. Using magnetencephalography in awake, resting subjects they provide evidence for widely distributed phase-amplitude coupling between respiration and different brain-intrinsic oscillations (delta, theta, alpha, beta, gamma). They further show that different frequency bands are differentially modulated at different locations, and that the identified networks have strong overlap with canonical networks, esp. the resting-state network.

The modulation of oscillatory brain activity by respiration has become a research focus during past years, resulting in a large body of literature from both, rodent and human studies. The observation that brain rhythms in humans are modulated by respiration is, hence, not entirely novel. However, the present article provides a very detailed, comprehensive picture of such modulations across brain regions and frequency bands. Moreover, the authors identified respiration-entrained sub-networks through clustering algorithms, and they map these networks onto known 'canonical' functional networks. The high resolution and degree of detail, the advanced methods of dimensionality reduction, the clustering and the mapping to the DNM are interesting and significant advances towards our previous knowledge.

We do appreciate the reviewer’s encouraging overall evaluation of our manuscript and will address their specific comments below.

As far as this reviewer can judge all methods have been applied very competently and methods and results are clearly laid out in the text and the illustrations. Nevertheless, there are some general concerns and several specific comments.

1. Nasal versus mouth breathing. Previous work by Tort and coworkers (mice, rats), Zelano and coworkers (humans) and others shows that, in most brain areas, respirational rhythms result from a feedback signal from nasal epithelia towards the brain. They are, hence, NOT caused by the rhythmogenic networks in the brainstem, but rather by sensory afferents (be it mechanosensitive or olfactory). It appears easy to test for the source of the described modulation by comparing the modulation of oscillations by breathing through the nose versus mouth (see, e.g., Zelano et al., 2016). I this reviewer's eyes, such a comparison would have introduced a clearly causal, rather than correlative finding into the paper. Without such (simple) manipulations, the work is mostly descriptive, though at a high level and, hence, still of great merit.

We thank the reviewer for pointing out the crucial distinction between nasal and medullar pathways in the generation (and propagation) of respiration-modulated oscillations. We wholeheartedly agree that these oscillations (which we termed RMBOs) are the result of mechanoreceptor signals (within the olfactory tract) being translated to higher-frequency neural oscillations, as we aimed to point out in the Discussion (kindly see section ‘Potential mechanisms behind RMBOs’). In contrast, the role of the brain stem first and foremost comes down to the generation of the respiratory rhythm itself, which is indeed not to be confused with the nasal pathway: During nasal inspiration, air enters the respiratory tract and triggers mechanoreceptors connected to the olfactory bulb, thereby initiating infraslow neural oscillations closely following the respiratory rhythm (*phase-phase coupling*). The phase of these slow oscillations then drives the amplitude of faster oscillations (*phase-amplitude coupling*) and propagates to upstream areas both within and beyond the olfactory system via medio-dorsal thalamic nuclei.
In fact, this well-described mechanism was the reason we continuously monitored participants during the measurement to ensure nasal breathing (see Methods), as multiple studies have demonstrated the absence of oscillatory modulations for oral breathing.
In order to strengthen the distinction between nasal and medullar pathways, we have amended both Introduction and Discussion which now include a more detailed description of the underlying mechanisms (also see our response to the reviewer’s related next comment). In addition, we have rephrased the brainstem-related paragraph in the Discussion to prevent any ambiguity in this regard:

[...] On the other hand, nasal respiration evokes feedback signalling in response to mechanical, thermal, or odor stimulation within olfactory areas in the forebrain, most prominently the olfactory bulb (OB) and piriform cortex (12). These (orbito-)frontal feedback signals are a central contributor for respiration-brain coupling, as animal studies have demonstrated that respiratory rhythms (i.e., air-driven mechanoreceptor signals within the OB) are translated into neural oscillations and propagated to upstream areas (13). (p. 2ff)

Cross-frequency coupling is widely regarded as the core mechanism of translating slow rhythms into faster oscillations and has conclusively been shown to be driven by respiratory rhythms within the OB in mice (13): During nasal inspiration, air enters the respiratory tract and triggers mechanoreceptors connected to the olfactory bulb, thereby initiating infraslow neural oscillations closely following the respiratory rhythm (*phase-phase coupling*). The phase of these slow oscillations then drives the amplitude of faster oscillations (*phase-amplitude coupling*) and propagates to upstream areas both within and beyond the olfactory system (46). (p. 11)

2. Interpretation of data. Related to the first point, the introduction and discussion over-emphasize brain stem, cerebellar and other caudal sources of respiration, while more frontal areas are mostly considered as motor-related networks. Thus, the literature on feedback signaling of mechanical, thermal and also olfactory cues is not sufficiently represented and discussed. Some parts of the discussion have been written in very global terms, e.g. on the importance of rhythmic activity for information processing, active sensing etc. It would be good to relate the concrete findings of the present work to these concepts and focus even more on what has actually been shown.

We appreciate the reviewer’s suggestions towards a more well-rounded write-up in the Introduction and Discussion sections. In addition to the revisions made in response to their previous comment, we have rephrased both sections as follows:

[...] Such fluctuating relationships between cortical networks could conceivably be modulated by changes in bodily states such as respiration. The full picture is complemented by two distinct pathways responsible for the feedforward generation of the respiratory rhythm and the neural processing of respiration-related signals, respectively: In addition to pattern generators like the preBötzinger complex in the medulla, deeper sites known to be involved in respiratory control comprise further subregions within the brainstem (18) and cerebellar nuclei (19). On the other hand, nasal respiration evokes feedback signalling in response to mechanical, thermal, or odor stimulation within olfactory areas in the forebrain, most prominently the olfactory bulb (OB) and piriform cortex (12). These (orbito-)frontal feedback signals are a central contributor for respiration-brain coupling, as animal studies have demonstrated that respiratory rhythms (i.e., air-driven mechanoreceptor signals within the OB) are translated into neural oscillations and propagated to upstream areas (13). (p. 2ff)

As the first olfactory relay station in the brain, the OB receives direct projections from receptors within the nasal cavity (36). These feedback signals not only encode odor information in olfactory receptor cells (37), but also mechanical stimulation of mechanoreceptors triggered by nasal airflow (38). As outlined above, subsequent neural activity patterns are then transmitted to upstream sites including OFC, hippocampus, and insula, which we fittingly found to be part of the RMBO network. (p. 10)

Furthermore, we have shortened the extensive discussion of subcortical sites as well as the overly general presentation of concepts like active sensing (kindly see Discussion section).

3. Explanation of methodological limitations. In EEG and MEG recordings, contributions from deep subcortical networks can mostly be indirectly assessed by complex algorithms which leave some room for uncertainty and interpretation. Many readers might not be familiar with methods of source localization in EEG/MEG recordings. While the technical procedures have been well described in the Methods section it would be very helpful to give a brief, qualitative account of the underlying principles in the main text. This could, e.g. be placed in Results (around Figure 2) and/or Discussion and address the underlying principles as well as the power and limitations of such methods.

We thank the reviewer for this suggestion as it will make the manuscript more accessible for non-experts. We have now included a short description in the Discussion section which reads as follows:

The voxel-based analysis employed adaptive beamforming for source localization. Adaptive beamforming optimally combines MEG recordings from all sensors to estimate the time series of neural activation at a given voxel while maximally suppressing interferences from other voxels. Although spatial resolution decreases with distance from sensors, it is generally suitable for cortical and subcortical areas. (p. 10)

4. Analysis of inter-regional interactions. In order to test whether oscillations of a given frequency are synchronized between two regions, coherence is a well-established measure. This reviewer does not understand why this relatively simple method has not been applied to the oscillations, revealing a frequency-resolved measure of coordination across frequency and regions. Such data would also substantiate the notion of distinct sub-networks by using an independent approach.

We fully agree with the reviewer that the investigation of synchronization between brain areas is highly relevant in this context. However, we believe that this analysis is beyond the scope of this manuscript for several reasons.

First and unfortunately, we can not simply compute coherence. Coherence as measure of interareal synchronization is sensitive to spurious zero-lag components that arise from imperfect source reconstruction (see e.g., Schoffelen & Gross, HumBrainMapp 2009; Bastos & Schoffelen, FrontSysNeurosci 2016). More complex measures such as Granger causality or weighted phase lag index need to be employed.

Second, such a synchronization analysis would greatly benefit from studying directional interactions (A->B versus B->A) that is, for example, available from Granger causality. This allows to more directly identify hierarchies within the cortico-subcortical respiration network. Again, this considerably adds further to the complexity of this analysis.

Third, connectivity analysis would ideally account for cross-frequency interactions that are particularly interesting in the context of respiration. We envisage scenarios (that have been observed in different contexts) where low frequency phase in one brain area modulates the amplitude in another brain area.

Fourth, connectivity analysis should be complemented with a comprehensive graph theory analysis that quantifies network properties using measures such as hub degree, centrality, efficiency etc.

We would like to note that we are in fact currently preparing a comprehensive connectivity-based investigation of respiration-brain coupling whose depth and complexity call for a separate manuscript.

Specific comments (not ranked by importance):

5. Fig. 1C seems to show a modulation of low-frequency MEG signals by respiration (visible at the bottom right corner of the panel). For better visibility it would be helpful to show the PTA-phase-relation over TWO, rather than one, respiration cycle.

We appreciate the reviewer’s remark and their suggestion to provide a more comprehensive visualisation of PTA effects. In fact, we were also eager to further explore the referenced positivity in the beta/low gamma range, but refrained from doing so due to the characteristics of the respiratory signal: Since the duration of respiratory cycles is variant even within a given participant, locking our analysis to a certain phase (in our case, peak inspiration, i.e. phase zero) is the only way to compute PTA and show within-cycle modulation. On the downside, this comes with increased noise around the edges of each respiration cycle, as is evident from the illustration below:

**Fig R1. Comparison of PTA over one (left) and two full respiratory cycles (right).** The panel is taken from Fig. 1C in the main text. Note that depicting PTA over the duration of multiple respiratory cycles comes with an increase in noise around the edges of each single cycle. This is due to between-cycle variance in duration (even within-subject) which limits the interpretability of effects approaching a respiration angle of ± pi.

Although we have a certain SNR decrease approaching the edges in Fig. 1C, we found this visualisation preferable to a PTA plot over two cycles. We hope that the reviewer can acknowledge this signal-inherent limitation.
For the sake of transparency, we have added a comment to the legend of Fig. 1 which reads as follows:

[...] Also note that SNR decreases towards the edges of the panel (i.e., approaching ± pi) due to increased variation of underlying single respiratory cycles which were used for phase-locked analysis. (p. 4)

As a final note, we would like to add that we are currently preparing a tutorial-style manuscript that outlines precisely how characteristics of the respiratory signal (such as within-/between-subject variability in duration or shape) determine methodological choices (e.g., phase-locked analyses) and which potential limitations they entail.

6. Upper graphs in in each panel of Fig. 2: the red horizontal line indicating significance threshold is barely visible in my version of the manuscript.

We thank the reviewer for raising this issue and have revised Fig. 2 accordingly.

7. For non-specialists, the large number of abbreviations makes the text difficult to read. Would it be possible to give full names upon the first mentioning of a technical / anatomical term within each section (i.e. for Introduction, Results, Discussion and Methods)?

Thank you, all abbreviations are now spelled out in full upon first mentioning within each main section.

8. The polar plots in Fig. 3 are not easy to read. As a minor correction, the authors might mark the 180° point as the point of inspiration (e.g., adding "I" or "In") and the 0° / 360° point as expiration. Moreover, it would probably be helpful to use a color code for the different frequencies, rather than grey scales.

We appreciate the reviewer’s suggestions regarding Fig. 3 and have revised the figure according to their recommendations.

**Reviewer #2, Jan Kujala:** The authors studied using MEG resting-state data how respiration influences neural signaling across the whole brain. The investigation was done via detailed spatio-temporo-spectral analyses. The results show that, globally, the whole spectrum of neural activity is affected by respiration. The results also show that different brain regions have distinct temporal and spectral profiles in how their activity is modulated as a function of the respiration phase. The results also suggest that these modulations have distinct spatial profiles where respiration is linked with low- and high frequency neural oscillations in lower and more lateral regions, respectively. The manuscript is well written and thorough and advanced analyses are conducted throughout the manuscript. However, I have some concerns whether the interpretations made based on the results are accurate. In particular, I find that additional analyses are required to demonstrate that the observed spatio-temporo-spectral specificity is meaningful instead of being an epiphenomenon of distinct profiles of neural activity and differential sensitivity of MEG in different brain regions.

We are thankful for the reviewer’s positive evaluation of our manuscript and will address his specific comments in detail below.

Specific comments

1. Globally, the results very clearly demonstrate that neural signaling across the whole investigated spectrum (2-150 Hz) is linked to respiration and the co-modulation is different across different frequencies. Moreover, the results show that these modulation spectra differ across brain regions. I am not, however, convinced that the present analyses demonstrate how meaningful the differences are and whether their relationship to oscillatory activity has a functional role. As the co-modulation of neural signaling with respiration seems to be very broad-band in nature as demonstrated by the authors as well as, e.g., Zeleno et al (cited by the authors), a strong possibility is that the observed temporo-spectral difference across brain regions are simply due to differences in the profiles of neural activity in them or due to how neural activity is detected with MEG. The same holds for the observed temporo-spectral specificity at the sensor-level. Namely, I find it possible that the majority of the findings within the manuscript could be explained, e.g., by a CO2-level dependent mechanism where all neural activity across the entire spectrum is influenced equally. If this holds, the observed differences could be due to the abovementioned distinct spectral profiles of neural activity across brain regions as well as due to differential delays in how CO2-levels follow the respiration phase in different brain regions. For the sensor-level results this explanation would mean that the spectral and temporal differences in the modulation index (Fig 1b) and phase-triggered average (Fig 1c) could represent the amount and/or SNR of neural activity across the entire brain and also the contribution of individual brain regions where a prominent relationship between neural activity and respiration can be detected due to these factors. Similarly, the voxel-level findings could be explained by variation in the detected spectral profiles of neural activity across brain regions as well as temporal differences in how the CO2-levels follow respiration. To dissociate between these possibilities (a more functionally meaningful role of the spatio-temporo-spectral specificity of the modulation vs. a simpler CO2-level dependent explanation), it would be valuable if the authors would, e.g., investigate the relationship between the modulation index and spectral profiles of neural activity both at the sensor-level and across different brain regions. A straightforward possibility would be to test how correlated the two measures are. If they would show only a weak link, it would suggest that there indeed is a functional role in the distinct spectral profiles of the co-modulation between respiration and neural activity, whereas a strong link between the measures would argue for a simpler explanation, e.g., a CO2-level dependent, global mechanism.

We thank the reviewer for providing such a detailed suggestion to control for ‘simpler’ (i.e. less specific) modulations. Following his remarks, we have now included a new control analysis in the Supplementary Material that is also referenced in the main text: Specifically, we used the output of the wavelet transform to compute voxelwise power spectra for each frequency used in our main analyses (i.e., 20,173 voxels x 36 frequencies). In keeping with our general analysis pipeline, we then restricted these whole-brain power spectra to component-specific maps, i.e. to those 202 suprathreshold voxels making up a given NMF component. For each of the 18 NMF components, power spectra were averaged across voxels, yielding one mean power spectrum per component per participant. Finally, for each component, we computed the Spearman correlation between each participant’s average power spectrum and their average MI spectrum, yielding 28 correlations x 18 components. To assess consistent positive relations between power and MI spectra, each component’s correlation coefficients were Fisher- z-transformed and subsequently tested against zero with a one-sided t-test. None of the components showed a significant group-level correlation between the two spectra (all p > .077 before multiple comparison correction). In our view, these results provide further evidence against a perfectly reasonable ‘simple’ mechanism (such as mere variation in CO2 levels) and in contrast argue for spectrally specific modulatory effects.

As mentioned above, we reference this new analysis in the main text and provide an in-depth explanation in the Supplementary Material:

Control analysis for spectrally unspecific modulations

In order to validate our interpretation of complex, frequency-specific modulations, we conducted a control analysis to investigate whether a more general, spectrally unspecific mechanism could equally explain our effects. Conceivably, such a mechanism could depend on CO_2_-levels and would merely relate respiration to ongoing oscillatory activity within brain regions. Any temporo-spectral differences across brain regions would then simply be due to differences in activation profiles of those regions, entirely independent of the differential respiratory modulation we propose.
To exclude such an alternative explanation, we tested to what extent the modulation spectra we observed were related to ongoing power spectra (independent of respiratory modulation). To this end, we used the output of the wavelet transform to compute voxelwise power spectra for each frequency used in our main analyses (i.e., 20,173 voxels x 36 frequencies). In keeping with our general analysis pipeline, we then restricted these whole-brain power spectra to component-specific maps, i.e. to those 202 suprathreshold voxels making up a given NMF component. For each of the 18 NMF components, power spectra were averaged across voxels, yielding one mean power spectrum per component per participant. Finally, for each component, we computed the Spearman correlation between each participant’s average power spectrum and their average MI spectrum, yielding 28 correlations x 18 components. To assess consistent positive relations between power and MI spectra, each component’s correlation coefficients were Fisher- z-transformed and subsequently tested against zero with a one-sided t-test. None of the components showed a significant group-level correlation between the two spectra (all p > .077 before multiple comparison correction). In our view, these results provide further evidence against a perfectly reasonable ‘simple’ mechanism (such as mere variation in CO_2_ levels) and in contrast argue for spectrally specific modulatory effects. (p. 23)

2. As regards the specificity of the findings across brain regions, I find that it would be beneficial if the analysis and illustration of non-negative matrix factorization based components would be expanded. Figure 2 demonstrates the spatial profiles of the components. However, the components are shown pooled together with lines indicating which cluster of voxels represents which component. It would be helpful if the component-specific spatial maps would be shown separately, either in the main manuscript or supplementary material. This would allow the readers to evaluate the whole set of voxels that primarily contribute to the component.

We thank the reviewer for his suggestion to include brain maps of single components for a better assessment of their spatial extent. Accordingly, the Supplementary Material now contains two new Supplementary Figures (Fig. S8 and S9) showing spatial maps of individual cortical and subcortical components, respectively:

**Supplementary Fig. S8. Individual spatial maps of cortical NMF components, projected on an inflated brain.** As in Fig. 2, whole-brain NMF components were thresholded at the 99th percentile, resulting in anatomical locations with an extent of n = 202 voxels.

**Supplementary Fig. S9. Individual spatial maps of subcortical NMF components, shown in frontal, sagittal, and transversal planes.** As in Fig. 2, whole-brain NMF components were thresholded at the 99th percentile, resulting in anatomical locations with an extent of n = 202 voxels. Crosshairs are positioned at the peak voxel of each location and atlas labels are provided (corresponding to the nomenclature in Fig. 2b).

Related to spatial profiles, it is unclear how the thresholding was done and what the results indicate. According to the manuscript the spatial maps were thresholded at the 99th percentile. Does this mean that they were thresholded at the 99th percentile across all components and voxels (18x20173)? That is, according the maximum value from amongst all voxels or components? Or was the thresholding component-specific? Or via some sort of a statistical evaluation as figure 2 states that "Colour bar indicates corresponding p-values"? If the component-specific maps would be shown in the manuscript it would be possible to show the unthresholded maps so that it would be possible to evaluate how spatially specific each component is.

Thank you for giving us the opportunity to clarify our thresholding procedure. After source reconstruction, we ended up with MI values for a total of 20,173 voxels inside the brain and, consequently, 20,173 entries in the coefficient matrix of each component (which quantified each voxel’s relative contribution to each of the 18 anatomical NMF components). As the vast majority of voxels contributed either very little or nothing at all to a particular anatomical component, we aimed to increase the signal-to-noise ratio for our subsequent analyses on the component level. Therefore, the threshold at the 99th percentile (across all 20,173 voxels for any given component) was chosen so that the resulting map would contain a reasonable number of voxels (in this case, 202 voxels), balancing specificity (i.e. rejecting non-informative voxels to increase SNR) and sensitivity (i.e. not neglecting the ROI’s spatial extension). The colour bar in Fig. 2 (as well as in the newly introduced Supplementary Figures) reflects the range of suprathreshold voxels and corresponds to p-values in normally distributed data. For clarity, we have revised the manuscript to consistently refer to percentiles only.

As mentioned above, we have now added component-specific maps as Supplementary Figures. In addition, we have clarified the anatomical thresholding on the component level in the Results and Methods sections:

Each component reflected respiration-modulated brain oscillations (RMBOs) across the frequency spectrum, quantified as NMF weights for each participant, voxel, and frequency. For spatial specificity of NMF components, each component’s spatial map was thresholded at the 99th percentile, yielding the n = 202 voxels with the strongest modulation. (p. 5)

Spatial maps were thresholded at the 99th percentile across all 20,173 voxels of each component, yielding the n = 202 voxels with the strongest modulation. (p. 6)

Spatial maps of all 18 network components are shown in Fig. 2. These maps were generated by thresholding full-brain maps (with a total of n = 20,173 voxels) at the 99th percentile, yielding spatially specific maps with an extent of n = 202 voxels. (p. 16)

Related to the spectral profiles of the components, it is unclear whether the modulation index values used in the statistical evaluation represent the relative average across all voxels or were modulation index values included only from the voxels that survived the 99th percentile thresholding.

Thank you for pointing out this missing detail. In response to the reviewer’s previous point, we have added further information concerning spatial thresholding to the manuscript (see above) which also addresses the present remark. To reiterate, statistical analyses were conducted on above-threshold voxels only in order to gain spatially specific results.

Regarding the spectral profiles of the components, it would also be beneficial to evaluate quantitatively how they differ between the components. At the moment, the manuscript presents for each component the frequencies where the modulation index is significant by comparing the actual data to surrogate data. In addition to this analysis, it could be evaluated, e.g., how individual components compare to the average of the other components. This could shed some light on whether the components are dissociated at specific, individual frequencies or whether the differences are more intricate with variation between component pairs in diminished and enhanced modulation index values across frequencies.

We welcome the reviewer’s suggestion to quantify spectral modulation between components. Following his remarks, the revised manuscript now contains an additional analysis that compares frequency-specific effects across the 18 NMF components: For all components *1..n* and each of the 36 frequencies used in our main analyses, we computed z-scores by comparing the MI value at frequency *i* of a given component *j* to the average MI value across the remaining 17 components:

$$z_{i,j}= \frac{\mu{MI}_{i,j} - \mu{MI}_{i, 1..n\backslash j}}{\sigma{MI}_{i, 1..n\backslash j}}$$

This yielded a matrix of 18 components x 36 frequencies quantifying the difference between each component’s MI spectrum relative to the grand average across all components. The newly introduced Supplementary Fig. S10 visualises this matrix thresholded at z = ± 2.33 (corresponding to p = .01):

**Supplementary Fig. S6. Comparison of frequency-specific modulations across components.** For each component and each frequency, we computed z-scores by comparing frequency-specific MI values of a particular component to the average across all other components (see main text for details). Opacity indicates significant differences (i.e., z = ± 2.33).

Here, a component’s MI values are considered significantly different from the mean of all other components when the difference exceeds the critical z value. Components #3 and #14 (both located within the left cerebellum) showed above-average modulation in the high gamma band, whereas components #6 (r. STG / r. temporal pole) and #11 (brainstem) were more strongly modulated in the delta and alpha band, respectively. Component #10 (bil. SMA) showed above-average modulation in the beta and low gamma range. Finally, component #12 (bil. ACC) was less strongly modulated at low gamma frequencies than the grand average across components.

This new analysis is referenced in the main text and outlined in detail in the Methods section:

In order to quantify modulation effects between components, we compared each component’s MI at a given frequency with the average MI across all other components. Components #3 and #14 (both located within the left cerebellum) showed above-average modulation in the high gamma band, whereas components #6 (r. STG / r. temporal pole) and #11 (brainstem) were more strongly modulated in the delta and alpha band, respectively. Component #10 (bil. SMA) showed above-average modulation in the beta and low gamma range. Finally, component #12 (bil. ACC) was less strongly modulated at low gamma frequencies than the grand average across components (see Supplementary Figure S6). (p. 5)

**Modulation differences across NMF components**

In order to compare modulation spectra across the RMBO network, we conducted a control analysis that compares frequency-specific effects across the 18 NMF components: For all components *1..n* and each of the 36 frequencies used in our main analyses, we computed z-scores by comparing the MI value at frequency *i* of a given component *j* to the average MI value across the remaining 17 components:

$$z_{i,j}= \frac{\mu{MI}_{i,j} - \mu{MI}_{i, 1..n\backslash j}}{\sigma{MI}_{i, 1..n\backslash j}}$$

This yielded a matrix of 18 components x 36 frequencies quantifying the difference between each component’s MI spectrum relative to the grand average across all components. Supplementary Fig. S6 visualises this matrix thresholded at z = ± 2.33 (corresponding to p = .01). A component’s MI values were considered significantly different from the mean of all other components when the difference exceeded the critical z value. (p. 17)

3. Related to both above points, the authors applied linear mixed effect models (LMEM) to quantify whether the observed apparent differences in the cluster-level spectral profiles according to the anatomical location of the clusters (Fig. 3) is significant. The results showed that the distance to the head centre influenced gamma band modulation whereas regions low in the sagittal plane demonstrated stronger modulation than high regions. However, as these were the only LMEM analyses that were conducted, it is unclear how specific these relationships are. The spatio-specral relationship within the modulation index could be explored further by, first, evaluating for gamma-band the influence of the height of the sagittal plane and for low frequencies the effects of the distance to the center of the head and, second, by investigating relationship along the other planes as well and not restricting the analyses to the apparent differences. Moreover, if the modulation index values are strongly linked to the profile of neural activity (point 1 above), the LMEM analyses could be conducted also for the spectral profiles of neural activity in addition to the modulation index spectra.

We welcome the reviewer’s proposal to extend the LMEM analyses of spatio-spectral modulation characteristics. Originally, in addition to investigating components’ distance to head centre, we focussed on the z plane as we reasoned it most appropriately distinguished cortical from deeper sites. We do, however, agree with the reviewer’s suggestion of a more comprehensive analysis and have therefore revised our manuscript as follows:
Within the main text, we focus our LMEM analysis on the distance to head centre. Following the reviewer’s remarks, these analyses are now provided for all six frequency bands. In short, we found RMBOs in the delta (t(502) = 3.66, p < .001) as well as the low (t(502) = 2.49, p = .013) and high gamma bands (t(502) = 3.85, p < .001) to significantly vary with components’ distance to head centre (kindly see the revised Results section for details). The remaining frequency bands showed no significant LMEM effects.
As the distance measure was computed as the vector norm from x, y, and z coordinates (see Methods), we extend the analysis of individual planes in the Supplementary Material. Here, we report frequency-specific LMEM analyses modelling individual contributions of the three planes, e.g.

MI_j_ = 𝛽_0_ + (𝛽_1_ + *S*_1j_) * *x* + (𝛽_2_ + *S*_2j_) * *y* + (𝛽_3_ + *S*_3j_) * *z +* *e*_j_

This way, as suggested by the reviewer, we can gain further insight as to how each plane contributed to the overall head centre effects and how spectrally specific these effects are. We provide a detailed description of the methodological approach as a new supplementary analysis (see Supplementary Material) and report the results in a new Supplementary Table S1:

**Supplementary Table S1. LMEM results for MI values as a function of x, y, and z planes.** Frequency bands were defined as described in the main text.

The plane-specific analyses show that the overall effect of distance to head centre on delta modulations can be traced to x and z coordinates, while low gamma modulations varied with z coordinates and high gamma modulations were dependent on coordinates of all three planes. Furthermore, we replicated previous results of specific z-plane effects for theta-band RMBOs and show that beta modulations specifically varied with x-plane coordinates.

The Abstract as well as the Results and Methods sections were revised according to the new analyses and now read as follows:

Globally, delta and gamma-band modulations varied with distance to the head centre, with stronger modulations at distal (vs central) cortical sites. (p. 1)

In order to investigate how MI spectra varied with anatomical location, we conducted a linear mixed effect model (LMEM) analysis modelling oscillatory modulations as a function of components’ distance to the head centre (considering x, y, and z planes). This analysis revealed that the fixed effect of distance to the head centre significantly influenced modulations within the delta (t(502) = 3.55, p < .001) as well as the low (t(502) = 2.49, p = .013) and high gamma bands (t(502) = 3.85, p < .001), with stronger modulations for more distal (compared to central) components. Further, frequency-specific analyses were conducted to characterise to what extent this overall distance effect was driven by sagittal, frontal, and transversal location, respectively (see Supplementary Material for details). (p. 8)

We employed linear mixed effect modelling (LMEM) to investigate the relationship between the spatial organisation and spectral characteristics within the network of modulated components. LMEM models a response variable (in our case, modulation indices within a particular frequency band) as a linear combination of fixed effects shared across the population (i.e. anatomical coordinates of network components) and participant-specific random effects (i.e modulatory variation between participants). To assess potential links between spatial and spectral component properties, we first computed each component’s anatomical distance to the head centre as the vector norm of MNI coordinates in the x, y, and z plane:

*r* = $\sqrt{x^{2} + y^{2} + z^{2}}$	(4)

We then specified an LMEM to predict modulation indices of a particular frequency band within each component as a function of its distance to the head centre:

MI_j_ = 𝛽_0_ + (𝛽_1_ + *S*_1j_) * *r* + *e*_j_	(5)

For participant j, the modulation index is expressed as a combination of the intercept (𝛽_0_), the fixed effect of the component’s distance to the head centre (𝛽_1_), and an error term (e_j_ ~ N(0,σ²)). We accounted for between-participant variation by specifying a random slope (S_1j_). An analogous approach was used to predict modulation indices within each component separately for each plane (see Supplementary Material). (p. 17)

As a final note, following the reviewer’s suggestion, we refrained from conducting additional LMEM analyses on power spectra since there was no meaningful link with MI spectra in any of the NMF components (see above).

Minor comments

1. In the methods section (p22, line 535) it reads that "adjacent significant data points were defined as clusters". As the data within the manuscript are multidimensional (spatio-temporo-spectral) it would be helpful to clarify whether the adjacency means spatial, spectral or spatiospectral adjacency.

Thank you, adjacency referred to the spectral domain; the respective paragraph has been clarified:

T-values were then thresholded at p = .05 and spectrally adjacent significant data points were defined as clusters. (p. 15)

2. In the methods section (p22, line 557) it is stated that "The sensor covariance matrix used for the LCMV-beamformer was computed across the whole data set.". I assume that this means that a single broad-band (2-150 Hz) beamformer was used to estimate the voxel-level activities across all frequencies. This could be clarified.

The reviewer’s assumption is exactly right, we have revised the Methods section for clarity:

A single broad-band (2-150 Hz) LCMV beamformer was used to estimate the voxel-level activities across all frequencies. (p. 15)

**Reviewer #3:** The authors describe here the anatomical location and spectral profile of the modulation of neural activity along the respiratory cycle, from human MEG data during resting state. While the issue is important and topical, I was left wondering what are exactly the results and whether all necessary controls had been performed. As detailed below, there is little validation of the (complex and novel) methods used, little discussion of possible confounds, but also some discrepancies in how the data look like or how they are described.

Major points

- The strength of MEG signals decreases with distance to sensors. Because the head moves with respiration, it follows that MEG signal power should vary across the respiratory cycle because of head movements. The authors provide a supplementary figure (supp Fig 7) illustrating the measured head movement with respiratory cycle and describe how they regressed out head motion (36 components) from the MEG data prior to data analysis. While this is certainly the best one can do with MEG data at the moment, whether this correction is sufficient or not is not known.

We thank the reviewer for acknowledging that our approach is ‘the best one can do with MEG data’ and we like to provide further analysis and arguments to convince them that this correction is sufficient. In response to their comments, we have amended the ‘Control analyses’ section of the Supplemental Information which illustrates our efforts to control for respiration-related head movements in more detail. Here, we present a comprehensive statistical assessment of linear and non-linear head motion regressors in relation to respiration-locked oscillatory changes. In short, while the respiration signal was significantly correlated to some traces of head movement, there was no significant relationship between head position/rotation and power across participants.
Specifically, we subjected a total of 36 GLM regression weights for each participant (2 movements [translation, rotation] x 3 directions [x, y, z] x 3 orders [first, second, third] x 2 levels [raw, derivatives]) to a group-level analysis: Individual regression weights were averaged across both runs, yielding a matrix of 36 regression weights x 20,173 voxels per participant. Applying the same cluster permutation approach we followed throughout our manuscript, we can confirm that there were indeed no clusters of voxels whose head movement regressors were significantly different from zero across participants.

There are several explanations why respiration-related head movements do not lead to systematic power changes at the group level.

First, head movements are individually different, both those that are related to respiration and those that are unrelated to respiration.

Second, not all head movements are expected to lead to power changes in the signal. Our voxel-level analysis utilises adaptive beamformers that are based on a linear combination of all sensor signals. Several movements (such as rotations, lateral movements, or movements along the posterior-anterior axis) will lead to simultaneous power increase in some sensors, but a decrease in others with small (or none) net power change.

Third, Supplementary Fig. S10 illustrates that respiration-related movements are very small (especially in comparison to other head movements such as the typical ‘sinking’ of participants across the duration of the measurement). This also explains why movement effects on power are not consistent across participants.

In summary, we have several reasons to believe that our correction is sufficient.

First, the regression removes effects from oscillatory power at the voxel level that can be explained by head movement (and derivatives up to third order). Consequently, our results are unlikely to be explained by head movement.

Second, our new group analysis indicates that regression weights were not significantly different from zero across participants in any brain area.

Third, head movement effects on power would lead to modulations that are spatially unspecific and evident across the full frequency spectrum. Instead, we see respiration-related modulations that are spatially localised and restricted to different frequency bands for different brain areas. This could potentially be explained by frequency-specific differences in signal-to-noise ratio. However, this interpretation would require a strong correlation between regionally specific power spectra and the respiration-related power modulation. Our new analysis shows that this is not the case (see our response to the first point raised by Reviewer 2).

- The results are described as follows in the Abstract: "high gamma modulation increased with distance to the head center, whereas delta and theta modulation decreased with height in the sagittal plane". I was left wondering whether such a physical, rather than physiological, description of the results corresponds to the fact that at least part of the results is due to the physical movement of the head with respiration, rather than to a true modulation of neural activity with respiration

This is closely related to the reviewer’s previous comment. To reiterate, there is considerable evidence against head movement being the source of modulatory effects: First, our regression analysis removed movement-related power effects (up to the third order) prior to MI computations. Second, regression weights were found not to be related to oscillatory power. Third, head movement would impact all frequencies equally and thus cannot cause the spectrally specific effects we report.
Kindly note, however, that we have revised the cited paragraph of the Abstract in response to the third comment made by Reviewer 2 (see above).

- This impression was reinforced by the fact that the authors did not attempt to demonstrate that they precisely replicate some of the known modulations of neural activity by respiration measured in animals or with intracranial EEG in humans. For instance, they could have a region of interest analysis in the piriform cortex (see Zhou et al eLife 2019 for a parcellation of the human olfactory system) to verify that they reproduce known findings.

Here, the reviewer continues to argue for their impression that head movement contributed to the reported modulatory effects. We have provided considerable evidence against that interpretation (including new computations) in our response to the reviewer’s previous two remarks (kindly see above). Finally, we would like to note that not only was our approach never intended to ‘precisely replicate’ previous findings (for numerous reasons outlined below), nor could the failure to replicate any result be considered evidence for the reviewer’s interpretation of movement-related artefacts. Animal studies aside, even a direct comparison with invasive human work e.g. from Zelano and colleagues (2016) would involve a variety of key differences seriously impacting the comparability of the two sets of results: First, the orientation of neural sources measured by EEG electrodes differs from those measured by our MEG system. Second, invasive recordings are by definition attached to a non-neurotypical brain. In case of epilepsy, for example, it is entirely unclear how differences in baseline excitability would affect RMBOs when compared to neurotypical brains (as in the present study).
Third, and maybe most importantly, intracranial recordings (as used in the Zelano study) necessarily record from fixed locations dependent on previous clinical assessment. Compared to the equally-distributed high spatial resolution of a whole-head MEG system, this introduces considerable differences in the statistical power of group-level modulation effects, as invasive recordings cannot equally cover anatomical regions (such as the piriform cortex) across patients. This is clearly evident from the leftmost panel of Fig. 4 from Zelano et al. (2016), where a) recordings are only available from 5 patients and b) there is substantial variance across participants, with e.g. gamma modulations only visible in a single patient. The key insight from this work, as the authors state themselves, was that nasal respiration entrains oscillatory activity in frequencies above the respiration frequency of about .25 Hz.
Given the rather inconsistent pattern of results within an already small sample, it is unclear what the ‘true’ to-be-replicated effect would even be. That being said, we were eager to provide the reviewer with some insight regarding present modulation effects within the piriform cortex. Below, we show respiration-locked time-frequency spectrograms gained from the piriform cortex of exemplary participants:

**Fig. R2. Time-frequency spectrograms from the piriform cortex (PC) of exemplary participants.** Analysis was conducted following the approach by Zelano et al. (2016).

Consistent with the findings by Zelano et al., some of our participants also showed PC modulation effects within the theta, beta, and gamma bands. Furthermore, it is evident that there is between-subjects variance with regard to the spectral as well as temporal characteristics of power modulations within PC, which is in line with the Zelano study.
While a group-level comparison between the two studies is inept for the reasons outlined above, we do hope that we have mitigated the reviewer’s concerns.

- The authors use a novel method, non-negative matrix factorization (NMF), which has been successfully used in other domains. However, this method has not been validated in the context of source localization of indices derived from MEG data. Could the authors provide some arguments / results to indicate that the results they present are not biased by the use NMF?

We thank the reviewer for the opportunity to provide further validation of the NMF approach with neural data. For example, Schoffelen and colleagues (PNAS, 2017) have provided a comprehensive account of NMF on source-localised MEG data and also compared the NMF analysis to a k-means approach (see the Supplementary Material of the referenced article). NMF has furthermore been employed in decoding analyses of M/EEG data (e.g., Delis et al., NeuroImage 2016) as well as in clinical resting-state applications (Phalen et al., Biol Psych 2020).

In response to the reviewer’s remarks, we have revised the Methods section to reference previous M/EEG applications of NMF:

NMF has previously been applied for topological analyses of M/EEG data during tasks (59), at rest (60), and in decoding approaches (61). (p. 15)

- Finally, the authors relate their results to arterial CO2 fluctuations during respiration (discussion, lines 405-415). Could it be that arterial impedance changes across the respiration cycle, leading to changes in the magnetic field and hence to the measured MEG data?

Much like the head movement-related changes the reviewer suggested earlier, any mechanism exclusively coupled to CO2 would uniformly influence oscillatory power across the entire frequency spectrum. Therefore, CO2 variation (or related impedance changes) alone cannot possibly explain the differential effects of respiration on oscillations of different frequencies. The reviewer’s remark is perfectly valid and was in fact raised by Reviewer 2 as well (see their first comment above). In response to their suggestions, we have conducted a new analysis specifically targeting spectrally unspecific changes over the respiration cycle (kindly see the Supplementary Material). In addition to the evidence presented above, this new analysis provides further support that the underlying mechanism is more specific than a mere coupling of oscillatory power to CO2 (or other spectrally unspecific changes related to respiration). The corresponding paragraph in the Discussion has been revised accordingly and now reads as follows:

On the one hand, respiration entrains OB activity via mechanoreceptors, as seen in local field potentials (25). On the other hand, Heck and colleagues propose extrabulbar sources within the brainstem which are of critical importance for the generation of the respiratory rhythm itself. Functionally, respiration thus appears to modulate higher oscillatory frequencies (e.g. gamma) for the purpose of integrating locally generated assemblies across the brain (51). Our data now show that respiration-brain coupling i) spans an even more extensive network including deep cerebello-thalamic pathways and ii) involves a wider variety of oscillatory modulation than previously assumed. Importantly, our analyses demonstrate that the spectral specificity of respiration-related modulations within the RMBO network cannot be explained by mere changes in CO_2_ alone. (p. 12)

Other concerns

- Could the authors indicate inspiration and expiration on their graphs? Does phase 0 correspond to lungs full or empty? Does it depend on where the respiration belt is placed?

We thank the reviewer for their remark concerning the readability of our figures. Phase 0 corresponds to peak inspiration, i.e. the maximum of lung capacity filled during the respiration cycle. Where applicable, all figures and figure legends have been revised to provide the necessary information As for the placement of the respiration belt, the exact placement is not that important, since differences in placement would only affect absolute values of thoracic circumference - these are normalised during preprocessing (see Methods). Nevertheless, our technicians aimed for consistent placement of the respiratory belt across participants (following manufacturer guidelines).

- There seems to be some discrepancy between the spectral profiles of MI (as shown in Fig2), that show peaks suggestive of frequency specificity, and the spectral profiles of component clusters, as shown in Fig3, that rather indicate a broad-band modulation. How come? For example, cluster C in Figure 3, composed of bilateral ACC and right FEF, has a spectral profile that looks quite different from the spectral profiles of bilateral ACC and right FEF as shown in Figure 2.

We appreciate the reviewer’s detailed comments on our figures. The discrepancy they note resulted from the way the normalised spectra were computed: While Fig. 2 was generated from the NMF’s basis matrix **A** (see Methods section for details), Fig. 3 was based on the ‘raw’ MI values per frequency x participant x component. Our rationale was to show NMF results and the corresponding z- and t-spectra together in Fig. 2, while the normalised spectra in the upper subpanels within Fig. 3 were merely intended to visualise the spectral similarities between the components that make up a given cluster. Originally, we intended to avoid redundancy in our figures (i.e., showing identical spectra in Figures 2 and 3). However, we are grateful that the reviewer has made us aware of this potential confusion and have accordingly revised Fig. 3 for clarity:

The spectra shown in each cluster’s top panel are now the same as in Fig. 2. As they are solely shown to visualise spectral similarity within each cluster, we have reduced the height of these panels. Phase information in the polar plots is now given in radians (see below). We believe the comparability between Figures 2 and 3 was further impeded by plotting log(frequencies) in Fig. 3, which has now been matched with Fig. 2. The figure legend has been revised to reflect these changes:

**Fig. 3. Time-frequency characteristics and anatomical distribution of component clusters.** Hind view of the glass brain illustrates the spatial distribution of component clusters A-G (numeration according to Fig. 2; see Supplementary Fig. S4 for top and side views). Spheres mark peak locations of components and are coloured according to cluster affiliation. Top curve plots depict z-transformed modulation indices of individual components within the cluster (group-level mean ± SEM, identical to Fig. 2) across all frequencies to visualise within-cluster similarities. Vertical bars mark borders between frequency bands (see Fig. 2 and main text). Polar plots show cluster-average RMBOs (colour-coded for frequency, see top left) as a function of respiration phase (where zero corresponds to the peak of the respiration signal). For clarity, polar plots are restricted to frequencies which were significantly modulated in at least one component of the respective cluster. Insp = inspiration, exp = expiration.

- The description of the results is sometimes contradictory. Figure 2 shows modulation of neural activity in visual regions (bilateral cuneus, bilateral lingual sulcus), but the authors say with the exception of SMA, all cortical modulations sites are non-sensory, belonging to the default, attention or saliency networks (lines 255-258).

We thank the reviewer for pointing out this oversight. The corresponding statement has been revised and now reads as follows:

Extending the distinction of deep vs more superficial components, cortical RMBOs were predominantly found in brain areas that have previously been established as nodes within the default mode network (DMN: PCC, angular gyrus, precuneus), dorsal attention network (DAN: FEF, aIPS), or saliency network (SN: insula, ACC; see 15). (p. 8)

Suggestions for improvement

- Fig 2, add some tick marks on the frequency axis, or write the value of the frequency for each vertical line

We appreciate the reviewer’s suggestion and have revised Fig. 2 accordingly:

- phases are sometimes expressed in degrees (eg Figure 3), sometimes in radians (eg Figure 4)

Thank you, the manuscript now consistently uses radians. This also affects Supplementary Fig. S5 which has been revised accordingly.

**Reviewer #4:** The study by Kluger and Gross investigates the relationship between respiration and different frequencies of resting-state cortical activity in a sample of healthy adults. As with any research question that tackles the relationship between brain measures and a metric that is traditionally seen as an artifact, I had concerns regarding the confounds of the respiration itself and the MEG data. Nonetheless, the authors are transparent about this and do a decent job of removing any confounds that may contaminate the data, which is unsurprising given the authors' expertise in signal processing. However, there seems to be a disconnect between the rationale of the study and the approach, which limits my enthusiasm for the manuscript. My specific comments are below:

Introduction/Discussion:

My biggest concern with this paper is the disconnect between the rationale of the paper and the research approach. The authors do a nice job of laying out the previous work in this field, including in animals and using iEEG. However, the justification for the role of the cortex in oscillatory modulation of respiration (or vice-versa) is that automatic respiration can be overridden using top-down mechanisms. Nonetheless, in the actual experiment participants are instructed to rest with their eyes open, and respiration is recorded passively. Given that there is no overt top-down modulation of respiration in the experiment, a conclusion related to these mechanisms is impossible.

We appreciate the reviewer’s overall assessment of our manuscript and we are eager to clarify the perceived disconnect they report. There appears to be a fundamental misconception with regard to the underlying rationale of the paper, which is why we would like to address two specific aspects of the reviewer’s comment:
First, the idea of cortical oscillations being modulated by respiration is by no means to be justified by mechanisms top-down respiratory control. While we certainly make the argument that top-down control of the breathing rhythm shows the bidirectionality between neural and respiratory signals (see Introduction), our study was essentially motivated by both animal (Ito et al., 2014; Yanovsky et al., 2014) and human work (e.g., Zelano et al., 2016; Herrero et al., 2018; Perl et al., 2019) showing that rhythmic brain activity outside of the olfactory tract is coupled to respiration. Most of the evidence in humans, however, has so far come from invasive recordings, which - despite their numerous merits - unfortunately do not allow a spatially comprehensive mapping of respiration-locked changes throughout the brain. Therefore, as we point out in the Introduction, we aimed to localise and characterise these modulatory effects at rest across the entire brain.
This is why, secondly, we explicitly refrained from including an overt top-down respiratory modulation in the present experiment. In fact, we have investigated the influence of voluntary deep vs normal breathing on oscillatory coupling in a previous study (Kluger & Gross, NeuroImage 2020) and discuss potential mechanisms there. In the present study, however, we were solely interested in passive, automatic breathing at rest and conducted our respiratory and MEG measurements accordingly.

Methods/Results:

What was the procedure for rejection of artifactual data outside of respiration/movement?

Besides the removal of respiration-related head motion and the control analysis regarding high-frequency muscle artefacts, no additional data were removed. Individual raw MEG data were visually inspected for jump artifacts and bad channels, but neither were detected. In a similar vein, individual respiration time courses were visually inspected for irregular breathing patterns such as breath holds or unusual breathing frequencies. Again, no artefacts were detected. We have amended the Methods section to include these details:

Individual respiration time courses were visually inspected for irregular breathing patterns such as breath holds or unusual breathing frequencies, but no artefacts were detected. (p. 13)

Individual raw MEG data were visually inspected for jump artifacts and bad channels, but neither were detected. (p. 13)

In your figures, can you please put corrected p-values rather than percentile? Additionally, the data seems to be thresholded at p = .01 corrected via permutation testing, which is a relatively liberal threshold (and judging from the maps, may be too liberal given the additional clusters that are shown but are likely spurious given their topographic distribution. What does the data look like at a more reasonable p = .005 corrected? Did you use a cluster correction and if so, what was the k?

We apologise for the apparent confusion caused by our description of the thresholding procedure. For the thresholding we used, stating the percentile is in fact the more precise value, as it should not be confused with a p-value resulting from a test statistic (which appeared to be the case for Reviewer 2, see above). In response to their comment addressing the thresholding procedure (see above), we have revised the respective paragraphs to make our approach more transparent:

Each component reflected respiration-modulated brain oscillations (RMBOs) across the frequency spectrum, quantified as NMF weights for each participant, voxel, and frequency. For spatial specificity of NMF components, each component’s spatial map was thresholded at the 99th percentile, yielding the n = 202 voxels with the strongest modulation. (p. 5)

Spatial maps were thresholded at the 99th percentile across all 20,173 voxels of each component, yielding the n = 202 voxels with the strongest modulation. (p. 6)

Spatial maps of all 18 network components are shown in Fig. 2. These maps were generated by thresholding full-brain maps (with a total of n = 20,173 voxels) at the 99th percentile, yielding spatially specific maps with an extent of n = 202 voxels. (p. 16)

As for the reviewer’s question regarding the topographic distribution of NMF components at a higher threshold, the below plot shows the MI spectrum for one exemplary component (in units of standard deviation, like the spectra shown in Fig. 2) for four different thresholds (left panel). While the SNR increase for the 99th percentile threshold (vs the 90th and 95th, respectively) is evident, further restriction of the component (e.g. thresholding at the 99.5th percentile) does not notably increase SNR but does reduce the number of voxels within the component, potentially neglecting more widespread effects. In order to balance sensitivity and specificity of subsequent analyses at the component level, we settled on a spatial thresholding at the 99th percentile. For illustration purposes, we also compare the anatomical extent of component #10 (bilateral SMA) thresholded at the 99th and 99.5th percentile (right panel):

**Fig. R3. MI spectra (left) and spatial extent (right) of an exemplary NMF component (#10) as a function of thresholding.** The NMF component was located within bilateral supplementary motor cortex (SMA). Left panel shows the component’s MI spectrum at different thresholds. Right panel shows a median view of the component’s spatial extent following thresholding at the 99th (top) and 99.5th percentile (bottom), respectively.

We hope that explaining our rationale and providing further information has mitigated the reviewer’s concern of our threshold being too liberal. To iterate, thresholding our whole-brain NMF components from n = 21,073 to n = 202 voxels is not comparable to e.g. thresholding fMRI activation maps at p = .01 (which would indeed be too liberal). In our case, significance of single components is not determined by their spatial extent (‘There is significant activation’ vs ‘There is nothing there’, which is what you would test for in fMRI). In contrast, the NMF yields 18 whole-brain components with different ‘weights’ given to each voxel, making them anatomically specific. Thresholding was merely used to narrow each component to those voxels with the largest weights, i.e. their anatomical centre. Significance was then determined on the frequency level by testing which oscillations were significantly modulated by respiration. This also relates to the reviewer’s next question concerning whole-brain vs ROI analyses, which we will address below.

I am confused as to your rationale for both a whole-brain and ROI approach. Please clarify.

Thank you, we gladly clarify the rationale behind our analyses. Overall, we aimed to structure the manuscript according to the underlying core questions, namely:

1. Does respiration globally modulate oscillatory brain activity?
2. If so, can we distinguish differential anatomical sources of these modulatory effects?
3. If we knew the anatomical sources of respiration-locked changes in oscillations, how are these modulatory effects characterised in time and frequency?

In order to answer these questions, it was important to start at the global, spatially unspecific level. Therefore, our initial whole-brain analyses (Fig. 1) were computed without any anatomical restrictions. Having answered the first question, we then proceeded to ‘zoom in’ on these global effects to see whether we could differentiate between distinct anatomical sources. To this end, for the second question, we extracted anatomical components underlying individual respiration-brain coupling. Critically, this analysis pipeline was entirely data-driven and therefore not at all a ROI-centred approach, as no regions of interest were a priori defined. We recognise that the word ‘ROI’ appeared once in our manuscript, and we apologize in case this caused any confusion. We have rephrased the respective paragraph accordingly:

We used the anatomical distribution of each network component (thresholded at the 99th percentile) to reduce this matrix to a component-specific spatial map and aggregated 36 single frequencies into frequency bands as follows: delta (2-4 Hz), theta (4 - 8 Hz), alpha (8-12 Hz), beta (12-30 Hz), low gamma (30-70 Hz), and high gamma (70-150 Hz). (p. 18)

Is there any concern regarding the perceived "direcitonality" of the effects, whereby the frequency changes decrease or increase as a function of distance from the respiration?

Directionality of effects is of great interest in our study and has at least two different meanings: First, there is a potentially ordered sequence of cortical events relative to the respiratory cycle. Second, there are likely causal directionality effects where one cortical event causes another. However, in our manuscript we are careful to avoid any discussion or interpretation of our results with regards to directionality since our current analysis is not suited to address these questions. It would require dedicated and sophisticated analysis that we are currently preparing for a separate study (kindly see our response to comment # 4 made by Reviewer 1).
