# Supplementary material for: Respiration modulates oscillatory neural network activity at rest
Source: PLoS Biol. 2021 Nov 11;19(11):e3001457. doi: 10.1371/journal.pbio.3001457 (PMC8610250; doi:10.1371/journal.pbio.3001457)
Supplement: Attachment — Submitted filename: REST_PLOS_R2_211013_Resp2Refs.docx [file pbio.3001457.s016.docx]

**Comments by the Editor**

1) Blurb: Please provide a blurb which (if accepted) will be included in our weekly and monthly Electronic Table of Contents, sent out to readers of PLOS Biology, and may be used to promote your article in social media. The blurb should be about 30-40 words long and is subject to editorial changes. It should, without exaggeration, entice people to read your manuscript. It should not be redundant with the title and should not contain acronyms or abbreviations. For examples, view our author guidelines: <https://journals.plos.org/plosbiology/s/revising-your-manuscript>

We appreciate the opportunity to provide a short summary of our work and provide the blurb in a separate document attached to our revision.

2) Ethics: Please include ID number of your protocol approved by the local ethics committee of the University of Muenster.

Thank you, the approval ID (2018-068-f-S) has been added to the manuscript.

3) Data: We note that you have stated that “The anonymised data supporting the findings of this study are openly available from the Open Science Framework (<https://osf.io/6zbdu/>).” However, the Data folder in the OSF link seems to be empty. Please double check and dismiss this request if I am wrong.

As noted in the resubmission form, the data were to be uploaded to the specified OSF folder upon acceptance of the manuscript. Following your positive evaluation, we have now made the data openly available via OSF. Within the main folder, the data are organised following the figure order of the manuscript which, together with the README files (see below) hopefully clarifies how each set of data was analysed to generate the respective figure.

3.1) Please also include a README file that explains how the data was analyzed to generate the following figure panels: Figures 1bc, 2ab, 3AG, 4d, S1, S5, S6, S9, and S10.

Thank you, README files have also been uploaded to the respective folders within the OSF directory (<https://osf.io/6zbdu/>).

3.2) Please also ensure that each figure legend in your manuscript includes information on where the underlying data can be found and that your supplemental data file/s has/have a legend.

We have amended the legends of all figures within the main text as well as in the Supplementary Material to reference the underlying data within the OSF folder (kindly see the revised manuscript file for details).
